# Orbital and suborbital temperature variability in the central Mediterranean across the Pliocene/Pleistocene transition

**DOI:** 10.1371/journal.pone.0310684

**Published:** 2024-12-26

**Authors:** Elena Zanola, Teresa Rodrigues, Sergio Bonomo, Patrizia Ferretti, Eliana Fornaciari, Agata Di Stefano, Alessandro Incarbona, Nereo Preto, Isabella Raffi, Luca Capraro

**Affiliations:** 1 Dipartimento di Geoscienze, Università degli Studi di Padova, Padova, Italy; 2 Divisão de Geologia e Georecursos Marinhos, Instituto Português do Mar e da Atmosfera (IPMA), Lisboa, Portugal; 3 Center of Marine Sciences, CCMAR, Algarve University, Faro, Portugal; 4 CNR- Istituto di Geologia Ambientale e Geoingegneria (IGAG), Area Della Ricerca di Roma, 1, Montelibretti, Italy; 5 Dipartimento di Scienze Ambientali, Informatica e Statistica, Università Ca’ Foscari di Venezia, Venezia, Italy; 6 Dipartimento di Scienze Biologiche, Geologiche e Ambientali, Università degli Studi di Catania, Catania, Italy; 7 Dipartimento di Scienze della Terra e del Mare, Palermo, Italy; 8 National Biodiversity Future Center (NBFC), Palermo, Italy; 9 International Research School of Planetology - IRSPS, Università degli Studi “G. D’Annunzio” di Chieti-Pescara, Pescara, Italy; Vanderbilt University, UNITED STATES OF AMERICA

## Abstract

A high-resolution record of central Mediterranean Sea Surface Temperatures (SSTs) based on the alkenone U^K’^_37_ index and planktic δ^18^O values for the surface-dweller *G*. *ruber* has been reconstructed across the Pliocene/Pleistocene transition at Monte San Nicola (Sicily), reference area for the GSSP (Global Boundary Stratotype Section and Point) of the Gelasian Stage. Spectral analyses indicate that the SST record is predominantly paced by a cyclicity in the ~47 kyr time domain, consistent with the obliquity driven glacial-interglacial variability that is expected to dominate in the interval of relevance. In addition, two suborbital periodicities in the ~5 kyr and ~8 kyr time domains provide a pervasive spectral signal that proves to be especially strong during the MIS (Marine Isotope Stage) 100 glacial, at the inception of the Northern Hemisphere Glaciation. This high frequency climatic instability, a prominent feature of the early Gelasian, might reflect episodic events of massive disruption of the Atlantic Meridional Overturning Circulation with increased production of cold, low-salinity water masses in the North Atlantic. Alternatively, it may be interpreted as the resonance (i.e., harmonics) of the low-latitude precessional forcing in mid-latitude regions. Although the driving mechanisms of these processes remain largely unconstrained, our study emphasizes the role of the central Mediterranean as the main reference for high-resolution paleoclimatic studies in the Neogene and the Quaternary.

## Introduction

The Piacenzian/Gelasian transition (P/G transition; ~2.6 Ma) has long been acknowledged as a crucial period in the recent evolution of Earth’s climate, as it marks the beginning of a global cooling that peaked with the definitive onset of Northern Hemisphere Glaciation (NHG; “Icehouse period” in [1]) in the early Gelasian.

Following a long interval of weak climate variability known as the “warm Pliocene”, referred to by many as a potential near-future analogue (i.e., [2]), the P/G transition developed through multiple cooling phases between ca. 2.8 and 2.4 Ma (namely, from MIS G1 to MIS 96), the aftermaths of which have been demonstrated to vary regionally. The establishment of large ice caps in the Northern Hemisphere is well documented in the North Atlantic marine sedimentary record, where the periodic deposition of Ice Rafted Detritus (IRD) was first detected at around 2.7 Ma [3]. The event is also marked by massive accumulation of loess in China since ca. 2.6 Ma, severe cooling in Northwestern Europe at around 2.5 Ma, and the influx of sub-Antarctic mollusks in New Zealand at ca. 2.4 Ma [4]. In the Mediterranean area, significant cooling events are reported to have occurred close to the base of the Gelasian Stage, at around 2.6 Ma [1, 5].

The NHG beginning is epitomized in δ^18^O records globally by a triplet of severe glacial periods (i.e., MIS 100 –MIS 98 –MIS 96; [6]), which provide a conspicuous marker for the event [7, 8].

In recent years the central Mediterranean region, particularly the Monte San Nicola area (MSN hereafter; Sicily, Southern Italy), where the GSSP of the Gelasian was established [9], has become the focus of integrated stratigraphic studies aimed at reconstructing the climatic and environmental responses of the central Mediterranean to the NHG onset and intensification [5, 10–13]. In addition to the expected orbitally-driven glacial-interglacial variability, ongoing investigations reveal a spectrally complex mixture of prominent cycles in the suborbital (i.e., millennial) time domain. So far, the short-term variability was only recognized based on the benthic record, initially for the MIS 100 glacial [11] and, more recently, for the interval between ca. 2632 and 2498 ka [13]. Here, we present the first high-resolution record of alkenone-derived SSTs and δ^18^O of *G*. *ruber* from a complete sedimentary sequence on the “Mandorlo” section of [12], located in the Western sector of MSN, providing evidence of the major climatic and environmental changes that occurred across the Piacenzian-Gelasian boundary.

## Materials and methods

**Fig 1:**
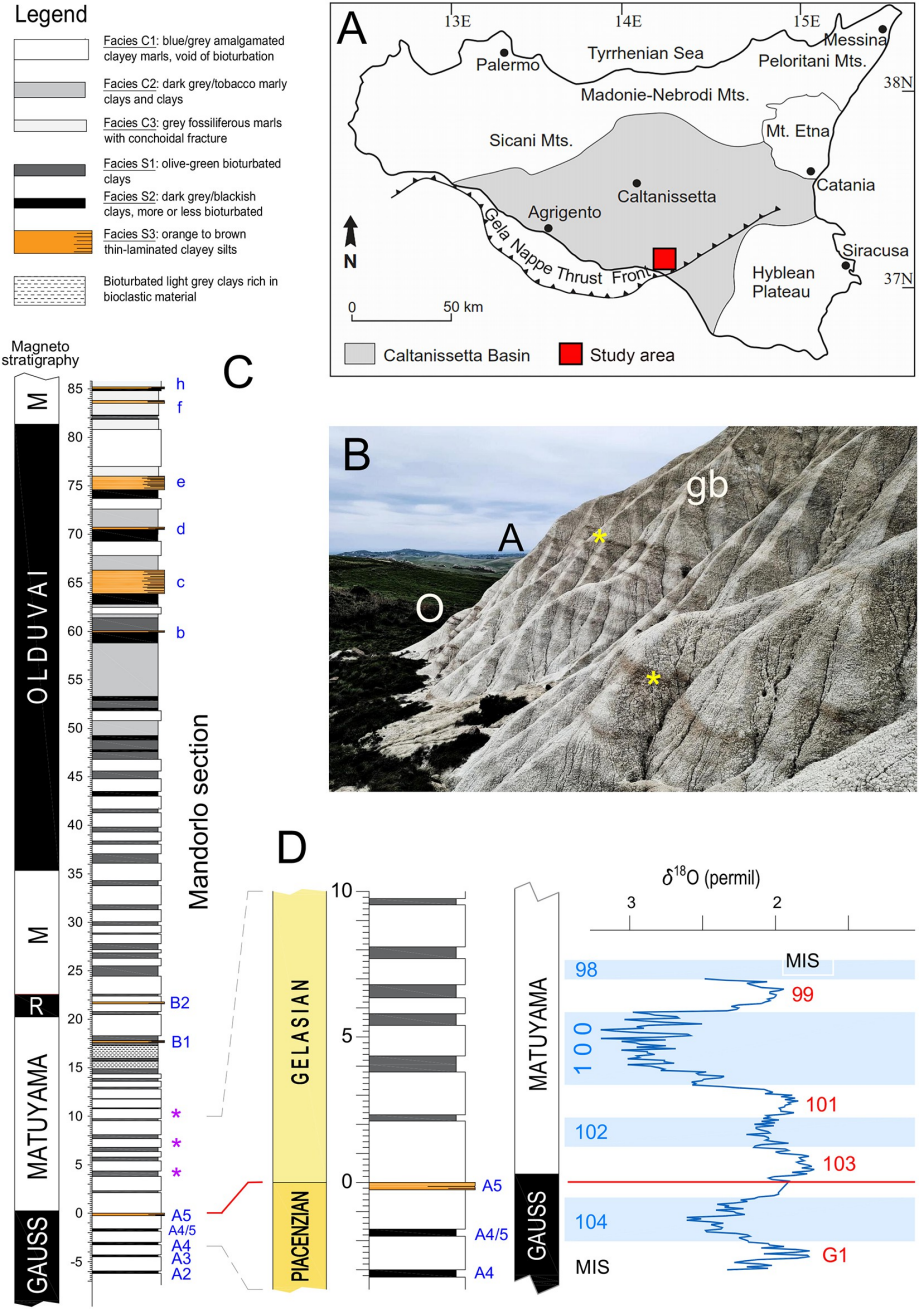
Geographical and geological context. A) Position of the Monte San Nicola area (red square) within the Caltanissetta sedimentary Basin (Sicily). B) Sapropel clusters O and A (upper Piacenzian) in the badlands close to the “Mandorlo” section. Yellow asterisks indicate the “Nicola bed” (sapropel A5), physical reference for the Piacenzian/Gelasian boundary and our reference zero level for sampling. The tripartite grey band (gb in figure) at the change of slope above the “Nicola bed” contains the MIS 100 –MIS 96 interval. C) Essential stratigraphic information for the “Mandorlo” section. Left: magneto-stratigraphy of section. R: Reunion; M: Matuyama. Right: lithological log. Legend is reported above. Individual sapropel layers of clusters A, B and C are labelled in blue. Purple stars: episodic influxes of left-coiled *Neogloboquadrina atlantica*, deemed as correlative to MIS 100, 98 and 96 (see [11]). D) Blow-up of the stratigraphic interval of relevance for this study. Left: chronostratigraphic, lithological, and magneto-stratigraphic logs (same as above). Right: the δ^18^O record for *Uvigerina* spp, with indication of glacial (blue) and interglacial (red) MISs. Light blue bands indicate glacial intervals. The thin orange line marks the Piacenzian/Gelasian boundary (Plio/Pleistocene as well). Modified from [13].

The area of interest is in the Western sector of the Monte San Nicola badland area, located in Southern Sicily, ca. 10 km north of the coastal town of Gela (Fig 1). The site offers a wide exposure of the upper Piacenzian to lower Calabrian stratigraphy, which consists of a ca. 80 m thick stack of open-marine muds [12]. Four individual clusters of Mediterranean Precession Related Sapropels (MPRS) are present, these being clusters O–A (ca. 3 to 2.6 Ma), and B–C (ca. 2.3 to 1.6 Ma; [14–16], which also allow for long-distance correlations.

For this work, we employed the sample set previously studied by [13], which was collected in the lower part of the “Mandorlo” section of [12]. The section, located close to the profile studied by 11], has been demonstrated to be more suitable and reliable for paleoclimatic and paleoceanographic investigations than the historical “Type” section, where the Gelasian GSSP was established [9, 17], which is poorly exposed and faulted in the interval above the Piacenzian/Gelasian boundary [12]. We analyzed a total of 200 samples collected with a steady sampling resolution of 5 cm along a 10 m-long profile by pinpointing the top of sapropel A5 (the so-called “Nicola bed”, marking the base of the Gelasian Stage) as our reference zero level. We extended our investigation downward to the top of sapropel A4 (-3 m, 2632 ka [13]) and upward to +7 m, reaching just above the dark clayey layer associated to i-cycle 242 (2498 ka [13]).

The age model used here is that previously presented in [13], which was assessed by linear interpolation among 9 tie-points provided by distinctive peaks in the δ^18^O record of *Uvigerina* spp. and/or sapropel layers (A2 –A5 [13]). Based on the estimated sediment accumulation rate of ca. 7 cm/kyr across this stratigraphic interval [13], our SST record is likely to allow for a temporal resolution of ca. 0.7 kyr, suitable to investigating the suborbital climate variability.

### C_37_ alkenones analysis and calibration

Alkenones are long-chain lipid compounds produced by coccolithophorid algae [18] that, in the Pleistocene, are believed to be mainly represented by *G*. *oceanica* and *G*. *caribbeanica* [19, 20].

They are synthesized with different unsaturation degrees based on local environmental conditions, being C_37:3_ and C_37:4_ typical of cold waters, and C_37:2_ abundant in warmer waters [21]. SST estimates can be obtained by means of a simple calibration equation [22].

For this study, alkenones were extracted following the standard procedure described by [23] based on [24], as described in S1 Text. Analyses were performed at the Bio-Geochemistry laboratory of the Instituto Português do Mar e da Atmosfera (IPMA) of Lisbon, using a SCION 435 Gas Chromatograph equipped with a Septum Programmable Injector (SPI) and a Flame Ionization Detector (FID). The analytical error is expected to generate an uncertainty in the SST estimates on the order of ±0.5°C and data replicability is extremely high, as the duplicate analysis of 20 random samples throughout the section yielded a negligible dispersion of the analytical results.

Among the numerous equations that are used to derive U^K’^_37_ SST estimates [22, 25–29], we opted for that of Müller [22] $\left(\frac{\left[C37:2\right]}{\left[C37:2\right]+\left[C37:3\right]}\right)$. because: 1) it is currently the most widely employed, thus best assessed, calibration method; 2) temperature estimates obtained using Müller’s equation proved to fit the measured SSTs in the modern Mediterranean basin [30–32]; 3) it allows for a direct comparison between our record and those previously reconstructed for the central Mediterranean [5], likewise based on Müller’s equation; 4) estimates obtained by means of Müller’s calibration are intermediate between those provided by [28, 29].

### Oxygen stable isotopes analysis

For δ^18^O analyses, 8 to 10 specimens of the planktic foraminifer *G*. *ruber* were hand-picked from the 250–350 μm size fraction and subjected to a cleaning treatment protocol [33] in order to remove any contaminant, such as clay or organic remains. Foraminifer tests were then crushed in glass vials, soaked in hydrogen peroxide (3%) for 30 minutes then soaked in acetone within an ultrasound bath for 30–60 seconds; finally, they were dried in oven at 50°C for 12 hours. All isotopic analyses were carried out at the isotopic ratio mass spectrometer laboratory of the Department of Geosciences of the University of Padova by means of a Thermo Scientific Delta V Advantage mass spectrometer equipped with an automated continuous flow Gas Bench II device. Sample acidification was performed at 70°C. Every 30 samples, an internal standard for quality control (Monzoni marble with δ^18^O values = -10.44 and δ^13^C values = 0.68) was measured; a second standard for calibration (Carrara marble with δ^18^O values = -1.15 and δ^13^C values = 2.58) was measured every 7 samples or less. The external error, derived by repeated measures of a quality control standard, is better than 0.10 ‰ for δ^18^O values. The isotopic results are expressed as permil (‰) on the VPDB scale.

### Spectral analysis

The analysis of non-stationary (frequency changes with time) and non-linear signals was performed on the SST record. Spectrum and wavelet images were produced both for the SSTs raw signal and for each of its identified relevant components (i.e., wave function), as well as for the planktic δ^18^O record (see S2 Text). This approach was conducted by applying the Ensemble Empirical Mode Decomposition algorithm (EEMD) by [34], which consists of an adaptive noise-assisted data analysis method, based on the assumption that any complicated signal can be decomposed into a finite, often small, number of components, also known as “Intrinsic Mode Functions” (IMFs) [35]. The decomposition technique represents a powerful method to study the different processes behind a given time series data and separates short time scale events (signal components) from a general trend, thus allowing for the recognition of signals in the sub-Milankovian time domain that are otherwise obscured by the dominant Milankovitch periodicity (e.g., obliquity). Each IMF represents an embedded characteristic simple oscillation on a separated timescale. IMF components were analyzed with “REDFIT” [36] and wavelet transform (wt). All data were detrended prior to running the spectral analysis, subtracting a linear regression line from the analyzed data. All analyses were carried out with R (version 4.1.3) using the Rlibeemd [37], dplR [38], and Biwavelet [39] packages.

## Results and discussion

### High-resolution SST evolution across the P/G boundary

**Fig 2:**
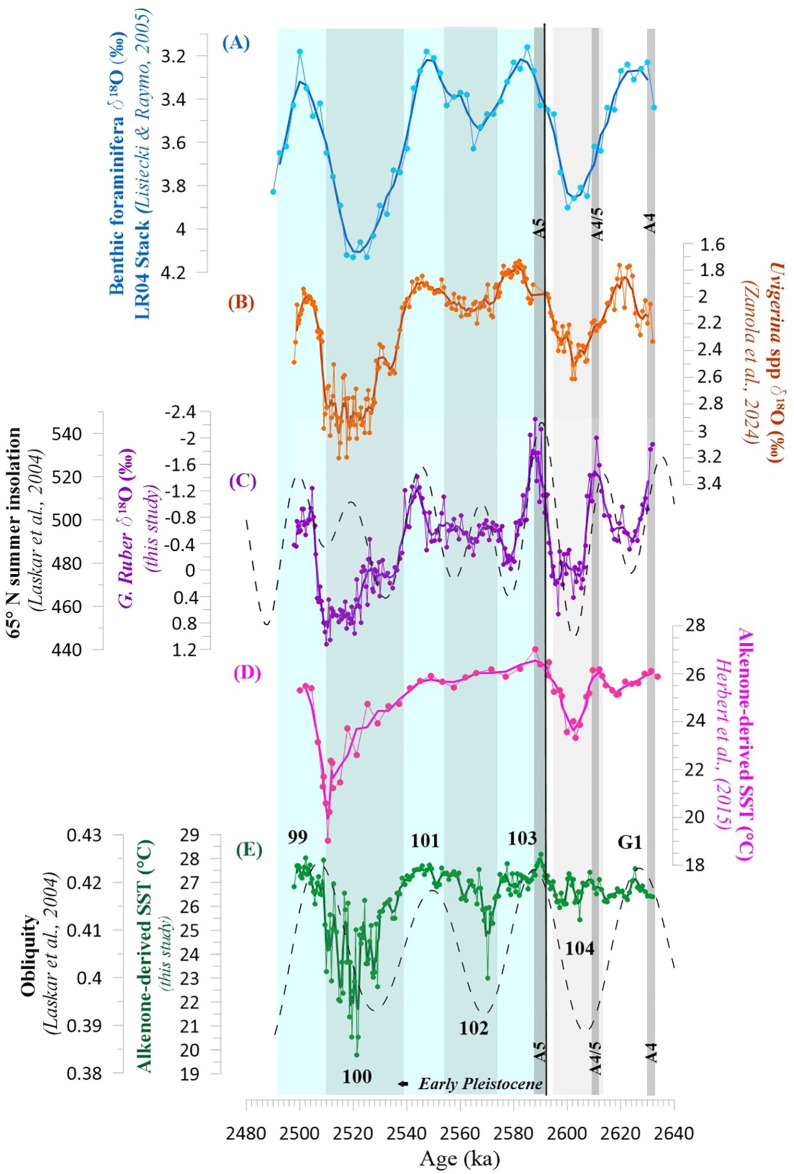
Paleoclimatic records for the “Mandorlo” section. From the top downwards: A) the LR04 benthic δ^18^O stack of [6]. B) the local δ^18^O record of *Uvigerina* spp. [13]. C) purple line: the high-resolution δ^18^O record for *G*. *ruber* reconstructed at the “Mandorlo” section (this study); dashed black line: 65° N summer insolation by [40]. D) the composite PP-MSN alkenone-derived Mediterranean SST record (°C) by [5]; PP = Punta Piccola and MSN = Monte San Nicola. E) the high-resolution alkenone-derived SST (°C) record for “Mandorlo” section (green) reconstructed for this study, based on the age model of [13] and plotted against the obliquity curve by [40] (dashed black line). Thick light gray bands indicate glacial stages (MIS 104, MIS 102, MIS 100). Thin dark gray bands indicate the sapropel layers of cluster A considered for this work (A4, A4/5 and A5).

SST estimates for the “Mandorlo” section are plotted in Fig 2. The mean U^K’^_37_ value in our record is 0.91, and alkenone-derived SSTs range between 19.8°C and 28.2°C. Long-term patterns in our record (Fig 2E) are in keeping with the oscillations documented for both the local and global benthic δ^18^O curves (Fig 2A and 2B, respectively), suggesting that regional SSTs across the interval of relevance were mainly driven by orbital obliquity. Between ca. 2632 ka (late MIS G2 interglacial) and ca. 2532 ka (late MIS 101 interglacial), SST values remained high (25°C to 28°C) with minor oscillations only (Fig 2E). The sole relevant exceptions are documented at ca. 2570 ka (early MIS 102 glacial), when temperatures decreased to ca. 23°C, and ca. 2540 ka (inception of MIS 100), when after a 6–7°C drop the central Mediterranean SSTs reached as low as 19–20°C. The latter event persisted for approximately 22 kyr and was characterized by a strong climatic instability, as demonstrated by six sub-millennial excursions in the SST record (Fig 2E). Synchronous, consistent oscillations are also documented in the local benthic δ^18^O record (Fig 2B; [11, 13]).

### Long-term (orbital) SST variability

The alkenone-derived SST record reconstructed for the “Mandorlo” section oscillate in keeping with the obliquity-driven glacial-interglacial variability, as described by the local benthic δ^18^O record ([13]; Fig 2B). As expected, glacial and interglacial periods correspond to lower and higher SSTs, respectively (Fig 2).

SST oscillations in the older part of our record (MIS G2 to early MIS 103; Fig 2E) are weak, suggesting that central Mediterranean temperatures barely responded to the Late Piacenzian glacial-interglacial variability. In correspondence with sapropel A5 (the “Nicola bed”) in MIS 103, temperatures attain a prominent maximum (~28°C) that persist up to ca. 2576 ka (Fig 2E). SSTs fall to ~23°C during the overlying MIS 102, which offers the first evidence of severe cooling in our record. Correlation with the SST reconstruction of [5] up to this stratigraphic level is not straightforward (Fig 2), possibly due to the different data resolution and age models (see S3 Text for discussion). Temperatures rose rapidly by 2–5°C at the beginning of interglacial MIS 101, followed by a ~14 kyr-long warm period (2554–2540 ka) with SSTs persistently close to 27°C, despite two weak cooling events before reaching the climate optimum at 2548 ka. The inception of MIS 100, commonly referred to as the NHG beginning [8], is marked by a severe drop in SSTs. For the first time in our record, Mediterranean surface temperatures fall below the 23°C threshold with a minimum of 19.8°C at ca. 2521 ka. Our SST record reveals that the long MIS 100 glaciation was characterized by an exceptional high-frequency climate variability, as testified by [11], similar to that documented for the last glacial period (MIS 2; [41, 42]). The termination of MIS 100 is marked by a stepwise increase in regional temperatures, culminating in the reestablishment of SSTs in the order of 27°C at the beginning of interglacial MIS 99 (ca. 2506–2497 ka).

**Fig 3:**
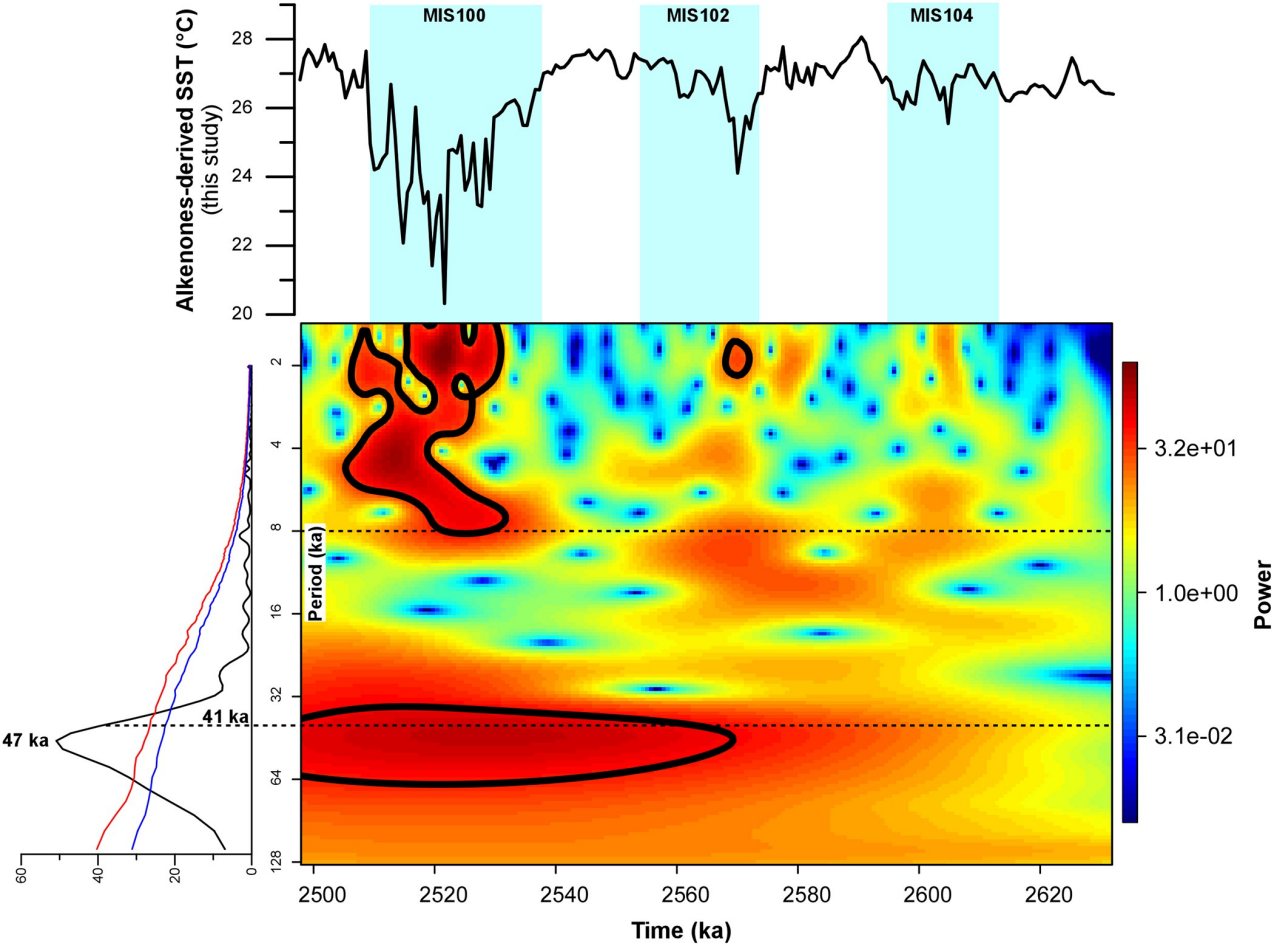
Spectral and wavelet analysis of the raw alkenone-derived SST signal. Bottom left: spectral analysis, revealing the presence of a 47-kyr signal (in the orbital obliquity time domain). Blue and red lines correspond to the 90% and 95% confidence thresholds, respectively. Bottom right: wavelet analysis. The colour scale is indicative of signal power. Red = stronger; blue = weaker. Top: our alkenone-derived SST record.

Spectral analysis performed on the SST raw signal data (Fig 3) unraveled the pervasive presence of a strong component with a ca. 47 kyr periodicity, thus suggesting—as previously noted by [5]—that the orbital obliquity forcing played a predominant role in driving the SST changes since the beginning of the Gelasian. This conclusion is further validated by the Wavelet record generated on the raw signal (Fig 3), showing that periodicities in the 32–40 kyr time domain became dominant beginning at ca. 2590 ka.

### Multi-proxy approach to sea surface conditions

The planktic δ^18^O record for *G*. *ruber* (Fig 2C) shows an array of pronounced oscillations that can be interpreted as a geochemical signature of the regional environmental and climatic variability. In agreement with the benthic δ^18^O record of *Uvigerina* spp., higher planktic δ^18^O values characterize glacial MIS 104 and MIS 100. Still, oscillations in the δ^18^O record of *G*. *ruber* proves to be largely independent of those documented in both the benthic δ^18^O and SST records (Fig 2). Records reconstructed for the central Mediterranean region (e.g., [43] and references within) prove that the isotopic record for *G*. *ruber* is predominantly affected by local hydrological conditions, such as the evaporation/precipitation ratio and freshwater budgets, which primarily respond to orbital precession (see Spectral analysis in S2 Text). Specifically, peaks of lower values in our δ^18^O_*G*.*ruber*_ record correspond to sapropel layers A5, A4/5 and A4, which formed under conditions of increased stratification of the water column in response to periods of massive precession-driven freshwater runoff [16, 44]. Our record confirms that the δ^18^O_*G*. *ruber*_ evolution in the Central Mediterranean is mainly shaped by precession-related changes in surface salinity, rather than glacioeustasy [45].

### SST fluctuations at the suborbital scale

**Fig 4:**
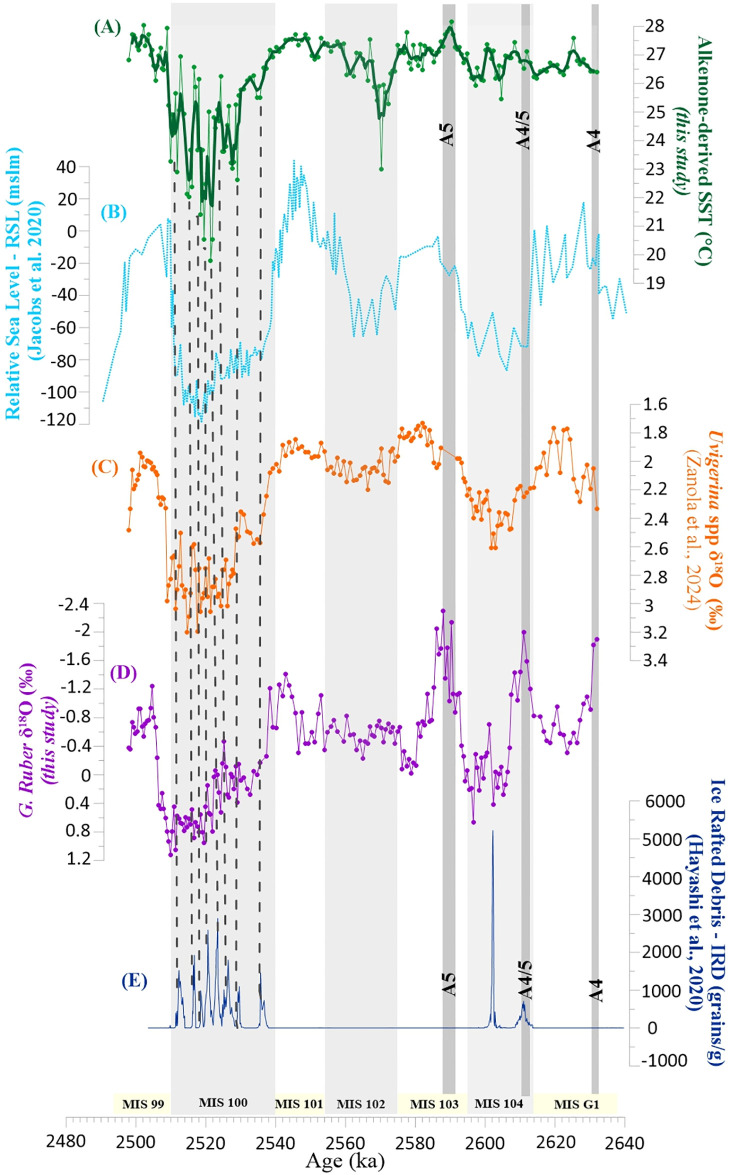
Climate variability at the suborbital scale. From the top downwards: A) the high-resolution alkenone-derived SST (°C) record for the “Mandorlo” section (this study). B) the Relative Sea Level (RSL) curve by [55]. C) the high-resolution δ^18^O record of *Uvigerina* spp. by [13]. D) the high-resolution δ^18^O record for *G*. *ruber* (this study). E) the Ice Rafted Debris (IRD) record by [3]. Isotopic values are reported in per mil (‰) relative to the VPDB standard. The dashed green lines mark the position of prominent IRD peaks within MIS 100. Thick light grey bands indicate glacial stages (MIS 104, MIS 102, MIS 100). Thin dark grey bands indicate the sapropel layers of cluster A considered for this work (A4, A4/5 and A5).

**Fig 5:**
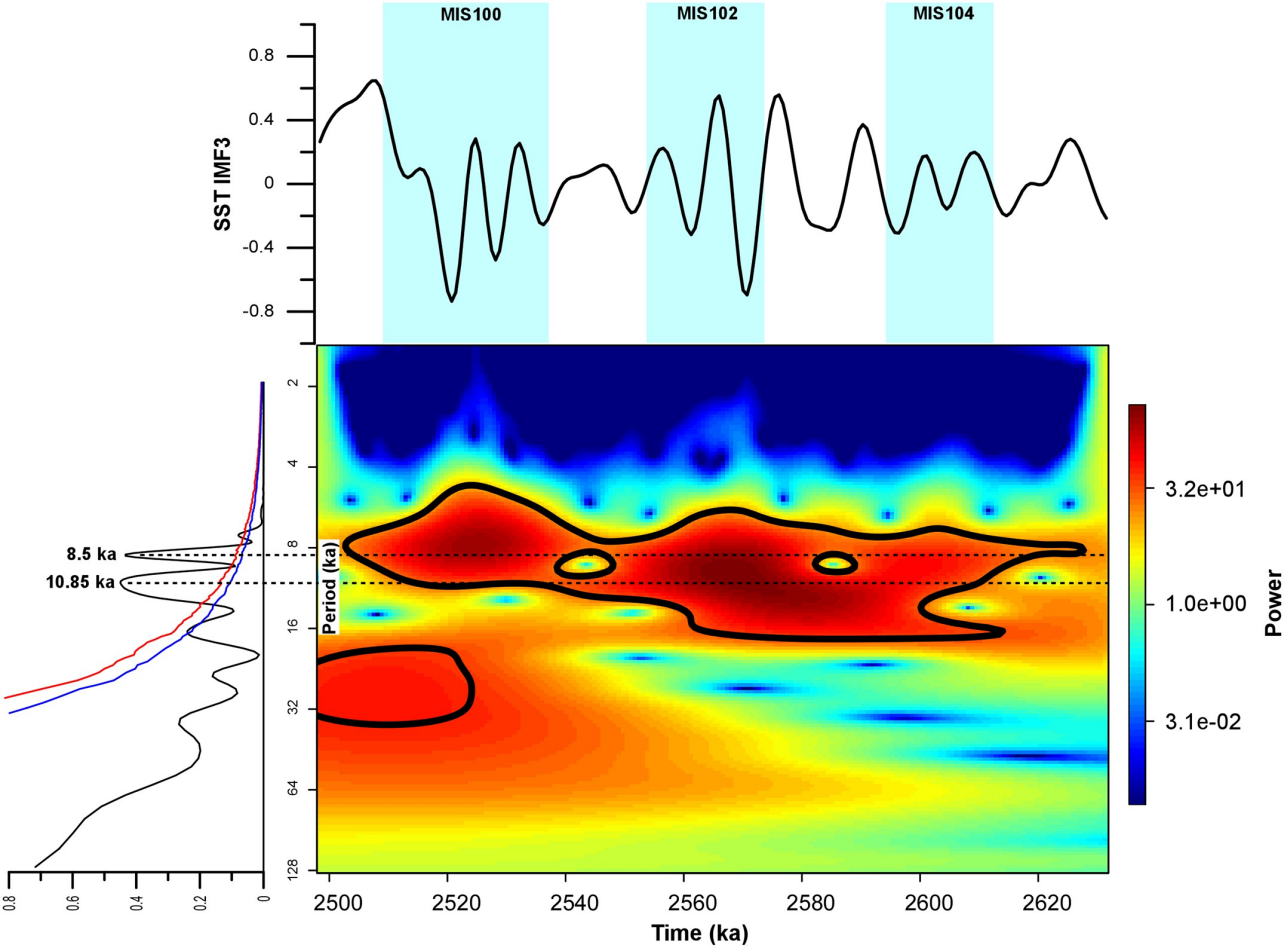
Spectral and wavelet analysis of the IMF3 component of the alkenone-derived SST record. Bottom left: spectral analysis revealing suborbital cycles on the order of 10.9 and 8.5 kyr. Blue and red lines correspond to the 90% and 95% confidence thresholds, respectively. Bottom right: wavelet analysis showing that the periodicities above are pervasive throughout the study interval, although they grow stronger since the beginning of the Pleistocene (signal power scale is reported on the far right: red = stronger, blue = weaker). Top: the SST IMF3 component spectrum.

**Fig 6:**
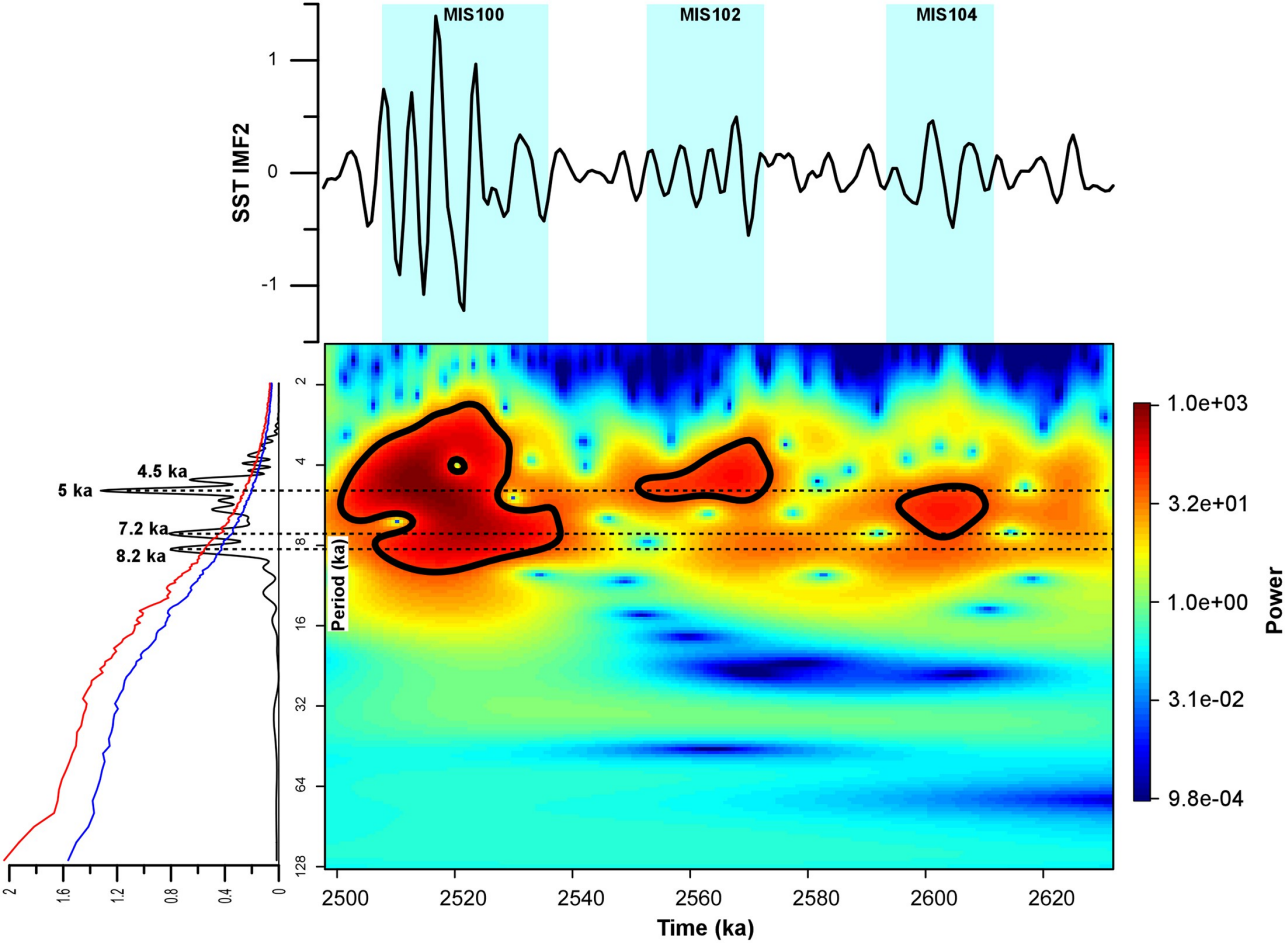
Spectral and wavelet analysis of the IMF2 component of the alkenone-derived SST record. Bottom left: spectral analysis revealing a second cluster of suborbital periodicities centred on the ~5 kyr band (i.e., 4.5, 5, 7.2 and 8.2 kyr, the 5 kyr period being the most significant). Blue and red lines correspond to the 90% and 95% confidence thresholds, respectively. Bottom right: wavelet analysis showing that said periodicities occur throughout but become stronger during the glacial stages, especially in MIS 100 (the signal power scale is reported on the far right: red = stronger, blue = weaker). Top: the SST IMF2 component spectrum.

Superimposed on the dominant orbitally-driven climate variability, the SST record presented here contains high-frequency oscillations that are generally more pronounced during glacial intervals (Fig 2A and 2E). In contrast with the overall temperature stability of the Late Pliocene and early Gelasian, several major oscillations in the SST record are found within the MIS 100 glacial (Figs 2E and 4A). This pattern is believed to mark the beginning of a strong millennial-scale climate instability across the Early Pleistocene glacial periods [46]. Spectral analyses revealed the presence of two main suborbital periodicities centered on the 10–6 kyr (IMF3 component) and 8–4 kyr (IMF2) band periodicities (Figs 5 and 6). In particular, the 10.9 kyr periodicity appears slightly more prominent than the 8.5 kyr, while the 5 kyr peak stands out as the most prominent within the 8–4 kyr band (Fig 6). Wavelet analyses computed for the IMF3 component confirm the persistence of a strong 10–6 kyr (~8 kyr) periodicity within the SST record (Fig 5). Fig 6 presents the signal power in the 8–4 kyr band (~5 kyr), showing similarities with the wavelet for the benthic δ^18^O record [13]. Both the SST and δ^18^O records show a prominent peak in the ~5 kyr band during MIS 100 that, to a lesser extent, is also well documented within glacial MIS 104 and MIS 102. In particular, the 5 kyr signal is best expressed within the MIS 104 –G1 interval for both the benthic and planktic δ^18^O records (Fig 4B and 4C), while it is centered on glacial MIS 104, MIS 102 and MIS 100 for the SST record (Fig 6). Evidence of a suborbital (millennial) climate variability with a 5–8 kyr periodicity at MSN, fully comparable to that recognized in the benthic δ^13^C and δ^18^O records of [13], was first reported by [11] for the interval straddling MIS 100 in a section adjacent to the “Mandorlo” section of [12] (Fig 4). More recently, an 8-kyr periodicity was also discovered in the coccolithophorid dataset from the same “Mandorlo” section [47], further emphasizing that this suborbital periodicity is a pervasive signal at MSN.

Periodicities in the 8–5 kyr band have been previously detected in various paleoclimatic records for the Last Glacial Maximum both in high-latitude regions of the Atlantic Ocean [48], mid-latitude areas of the Pacific [49], and low-latitude sectors of the Mediterranean Sea [11, 50, 51]. [49] unraveled a periodicity of ~8 kyr in the alkenone-derived SST record of the Okhotsk Sea over the past 120 kyr, which was correlated to the dynamics of the Pacific Climate Index (PCI). [51] report on comparable cyclicities (~5 and 8 kyr) over the last 50 kyr. They consist in abundance fluctuations of *N*. *pachyderma* (SST proxy) and steppe pollen (aridity proxy) in the Western Mediterranean [52], which are interpreted as correlative to Henrich Events in the North Atlantic, and oscillations in the *n*-*hexacosanol/n-nonacosane* (AP) index (proxy of deep-water ventilation; [50]), tracking the Greenland PCI index (i.e., changes in the intensity of the high-latitude atmospheric circulation).

[50, 51] explained the occurrence of suborbital signals in the Mediterranean across the last glacial period as the regional response to changes in the strength of the AMOC (Atlantic Meridional Overturning Circulation) upon the NHG intensification.

Previous works interpreted the high-frequency climatic events identified in the Late Pliocene and Early Pleistocene as analogues of the Late Pleistocene Dansgaard-Oeschger events and the Holocene Bond cycles [53]. More specifically, [54] demonstrated that the Henrich-like events found in Site U1313 (North Atlantic) between 960 and 320 ka correlate with episodes of ice calving from the Laurentide Ice Sheet and icesheet accretion (i.e., NHG intensification) at the end of the Mid-Pleistocene Transition. A similar mechanism was invoked by [11] for explaining the high-frequency oscillations found in the benthic δ^18^O record of MIS 100 at MSN, as later confirmed by North Atlantic sedimentary records ([3, 55]; Fig 4E and 4B. Finally, the record of *Florisphaera profunda* at MSN by [47] shows numerous oscillations within MIS 100 that are in phase with both the record of IRD deposition in the North Atlantic and the “stadial” episodes found in the SST record presented here.

Orbital variations may impact suborbital changes through rapid processes, such as sea ice dynamics, as well as through slower changes in ice sheet volume and, consequently, eustasy [46]. Late Pliocene and Early Pleistocene glaciations were characterized by decreasing temperatures and increasing productivity in surface waters of the North Atlantic [56], suggesting that the North Atlantic Current (NAC) weakened and the Artic Front (AC) underwent a southward migration. During periods of intensified NHG, the NAC was likely subjected to predominant east-west flows, with limited meridional heat transport and cooling at higher latitudes. The overgrowth and destabilization of continental ice caps in the Northern Hemisphere promoted periods of massive iceberg rafting and amplification of the climate variability at the millennial (suborbital) time scale, which we deem responsible for the short-term palaeotemperature fluctuations observed at MSN.

Global Circulation Models for the last glacial periods suggest that events of AMOC slowdown/shutdown would promote cooling throughout the whole Northern Hemisphere [57, 58], including the Mediterranean basin, while the Polar Circulation Index would strengthen the atmospheric circulation over the region [50, 51, 59]. Accordingly, the AMOC can be identified as an effective mechanism for suborbital climate regulation at the hemispheric scale, even in the earliest stages of NHG. Short-term cooling/warming events (“Millennial Climate Variability” in [46]) may be the result of multiple interactions of internal/external forcing, such as direct effects of insolation, increase/decrease in continental ice volume, changes in sea level and, consequently, rapid changes in the AMOC strength [60].

Still, suborbital climatic oscillations recognized in Atlantic records such as the IODP Sites U1313 (North Atlantic; [61, 62]) and U1385 (Iberian margin; [63, 64]), have been interpreted as the response to different source mechanisms than those discussed above. In fact, millennial-scale climate instability in mid-latitude regions might have been promoted by precession-related insolation changes in low-latitude areas echoing/resonating back as harmonics of the main ~21 kyr signal (second and fourth harmonics, respectively; in our case, ~10.9 and ~5 kyr), in keeping with the periodicities found at MSN. Considering the available information, both the models discussed above are believable, and further independent data will be necessary to settle the matter.

## Conclusions

The ~134 kyr long paleoclimate records presented here allowed reconstructing the first high-resolution paleotemperature record across the Piacenzian-Gelasian boundary for the central Mediterranean region. By comparison to the local benthic δ^18^O record, it was established that the main oscillations documented in our SST record reflect the obliquity-driven glacial-interglacial climate variability that is known to dominate throughout the interval of relevance. In addition, spectral analyses enabled the identification of suborbital oscillations, among which two (namely, ~5 and ~8 kyr) were detected in many paleoclimatic records for both the Mediterranean and the open ocean [49, 51, 54, 60]. The interval corresponding to the MIS 100 glaciation is characterized by an unprecedented collapse in Mediterranean SSTs and a strong climatic instability within. Here, a prominent signal was detected of SST fluctuations in the ~5 kyr time domain, which may either reflect the dynamics of the North Atlantic in response to the periodic overgrowth and destabilization of Arctic icecaps, with temporary disruption of the AMOC, or the regional response to precession-driven subtropical processes. The available data cannot provide a conclusive response to the matter, and further research will be necessary to elucidate the very origins of the suborbital cycles that are documented to occur across the Early Pleistocene interval.

## Supporting information

S1 Text Analytical procedures for alkenone extraction. (DOCX)

S2 Text Spectral analysis on the δ^18^O record of *G*. *ruber*. (DOCX)

S3 Text Correlation to the alkenone-derived SST record of [5]. (DOCX)

S1 File Inclusivity in global research. (DOCX)
