# Supplementary material for: Orbital and suborbital temperature variability in the central Mediterranean across the Pliocene/Pleistocene transition
Source: PLoS One. 2024 Dec 26;19(12):e0310684. doi: 10.1371/journal.pone.0310684 (PMC11671011; doi:10.1371/journal.pone.0310684)
Supplement: S1 Text — (DOCX) [file pone.0310684.s001.docx]

**S1 – Analytical procedures for alkenone extraction**

For alkenone extraction, dry sediment samples were manually ground, transferred into tubes and weighed for an amount of circa 2,5 g. After the addition of an internal standard mixture containing n-nonadecan-1-ol (C_19_-OH), n-hexatriacontane (C_36_) and n-tetracontane (C_40_), dry sediments were subjected to a series of extractions with dichloromethane in an ultrasonic bath. Extracts were then purified by hydrolyzation with 6% potassium hydroxide in methanol for removing wax esters, and fatty acids (saponification). Organic compounds were recovered with hexane and evaporated to dryness with an N_2_ stream. Ultimately, the extracts were redissolved with toluene and derivatized with bis(trimethylsilyl)trifluoroacetamide (BSFTA) until analysis.

A CPSIL-5CB capillary column (50 m length, 0.32 mm internal diameter and 0.12 µm stationary phase thickness) was employed for compounds separation. Hydrogen was the carrier gas (with a flow rate of 2.5 ml/min). The oven temperature was programmed from 90 °C to 170°C at 20 °C/min, then to 280 °C at 6 °C/min (holding time of 25 min) and finally to 315 °C at 10 °C/min (holding time of 12 min). The injector was programmed from 90ºC (holding time 0.3 min) to 310 ºC at 200 ºC/min (final holding time of 58 min). The detector was set at constant temperature of 320 ºC.

Alkenones concentrations were determined by comparison of peak areas with those of n-hexatriacontane peak used as an internal standard, using the following formula:

[Biomarker] ng/g = (biomarker area/ C_36_ area) * (ng C_36_/dry weight (g))

Afterwards, SST was estimated using the alkenones U^K’^_37_ index $(\frac{\left[ C37:2 \right]}{\left[ C37:2 \right]+\left[ C37:3 \right]})$, which is a simplified version of the U^K^_37_ index, being based only on the di- (C_37:2_) and tri-unsaturated (C_37:3_) alkenones ratio [1]. The cold-water form SST estimation were calculated based on the global core top calibration of annual SST [2] with an uncertainty of 1.5 ºC. Calibration equation used here is thoroughly discussed in section *“Materials and methods”*. Percentages of recovery were calculated on the basis of an internal standard and a blank, in order to check the performance of the analytical method. In most cases, recovery was close to 100%.

**References**

1. Prahl FG, Wakeham SG. Calibration of unsaturation patterns in long-chain ketone compositions for palaeotemperature assessment. Nature. novembre 1987;330(6146):367–9.

2. Müller PJ, Kirst G, Ruhland G, Von Storch I, Rosell-Melé A. Calibration of the alkenone paleotemperature index U37K′ based on core-tops from the eastern South Atlantic and the global ocean (60°N-60°S). Geochimica et Cosmochimica Acta. maggio 1998;62(10):1757–72.
