# Supplementary material for: Orbital and suborbital temperature variability in the central Mediterranean across the Pliocene/Pleistocene transition
Source: PLoS One. 2024 Dec 26;19(12):e0310684. doi: 10.1371/journal.pone.0310684 (PMC11671011; doi:10.1371/journal.pone.0310684)
Supplement: S2 Text — (DOCX) [file pone.0310684.s002.docx]

**S2 – Spectral analysis on the δ^18^O record of *G. ruber***

For the sake of consistency, spectral analyses were also conducted on the δ^18^O of *G. ruber.* Three major IMF components were found: IMF4 with a ca. 46.2 kyr period (obliquity domain); IMF3 with ca. 22.1 kyr period (precession domain); IMF2 characterized by major cyclicity centered at ca. 9.6 kyr. Wavelets generated on the IMFs components signal further validate the previous observation, showing that orbital periodicities in the 46.2 kyr and 22.1 kyr order are pervasive and continuous throughout the entire time interval. Likewise, the sub-orbital 9.6 kyr cyclicity seems to be continuous but slightly less pervasive.


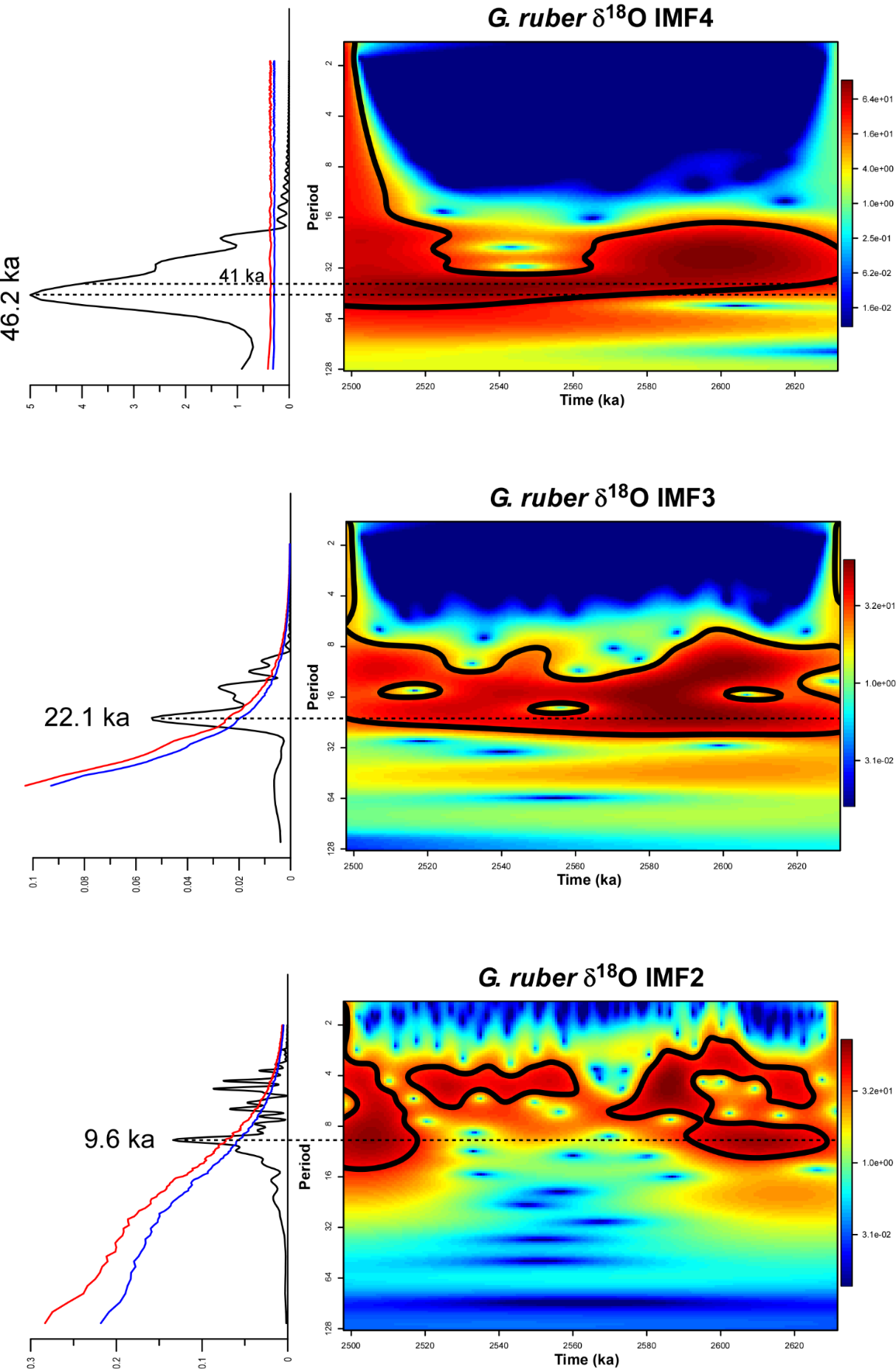


**Figure S2:** Spectrum and wavelets for the three prominent IMPS signal components: IMF4 (46.2 kyr cyclicity; obliquity domain), IMF3 (22.1 kyr cyclicity; precession domain) and IMF2 (9.8 kyr cyclicity).
