# Supplementary material for: Orbital and suborbital temperature variability in the central Mediterranean across the Pliocene/Pleistocene transition
Source: PLoS One. 2024 Dec 26;19(12):e0310684. doi: 10.1371/journal.pone.0310684 (PMC11671011; doi:10.1371/journal.pone.0310684)
Supplement: S3 Text — (DOCX) [file pone.0310684.s003.docx]

**S3 – Correlation to the alkenone-derived SST record of [1]**

A composite section reconstructed by merging the “type” MSN section and the coeval part of the Punta Piccola section (Southern Sicily) was employed by [1] in 2015 for establishing a long Mediterranean SST record across the Pliocene-Pleistocene transition. Comparison between our high-resolution record and the curve of [1] reveals important discrepancies in the stratigraphic interval immediately below MIS 100. In particular, the record of [1] shows a major SST decrease in correspondence to the interval interpreted as MIS 104, while no oscillations occur in MIS 102. This pattern in full contrast with our record (Fig. 2 in the main article). Although these differences may simply depend on the higher time resolution of our record, other explanations are also possible. First, we stress that our previous investigations in the very same “type” MSN section exploited by [1] demonstrated that the stratigraphic interval immediately above the Gelasian GSSP is affected by several problems, such as the bad exposure conditions due to creeping and vegetation (which make it impossible to sample along a single continuous profile), as well as the presence of a thick network of faults that are likely to delete and/or duplicate part of the record. Secondly, the age model used by [1] is based on the direct correlation between peaks of C_37_ abundance and maxima in the monsoon index of [2] while our chronology depends on physical stratigraphic and bio-magnetostratigraphic markers. To test the consistency of the two records, we have attempted redrawing the curve of [1] by imposing that their MIS 104 represents MIS 102 instead and employing the age model of [3]. The -0.8 °C correction in the MSN-PP composite record used by [1] was removed, because different SST values can coexist offshore southern Sicily due to the presence of semi-permanent meso-scale cyclonic and anticyclonic gyres, like in today’s Adventure Bank Vortex and Maltese Crest Channel [4,5]. The result (Fig. S3) is impressive, as the oscillations documented in both records are almost identical in terms of shape and amplitude. We conclude that most likely, the discrepancies between our record and that of [1] is due to the use of different age models.


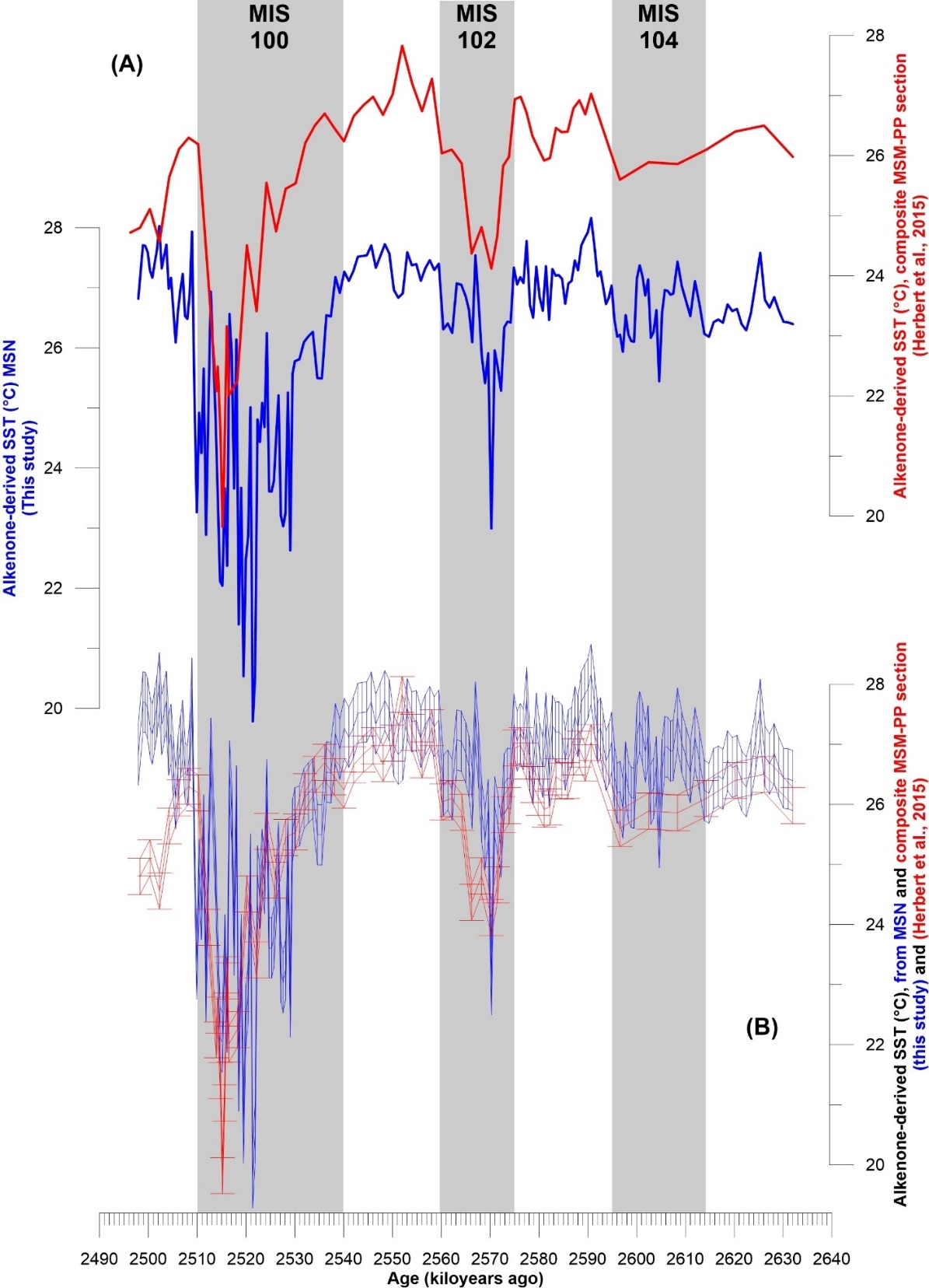


**Figure S3**: Comparison between the alkenone-derived SST records for the “Mandorlo” section (this study) and the Monte San Nicola-Punta Piccola section (MSN-PP) of [1].

Panel A: SST data from the “Mandorlo” section (blue line) and the MSN-PP composite record (red line), plotted on a common y-axis (SST °C), are aligned supposing that the cooling interpreted as MIS 104 by [1] corresponds to MIS 102 instead. The only relevant mismatch is between 2510 and 2500 ka (top of the record), where the age model of [3] becomes less accurate due to the lack of relevant bio-magnetostratigraphic and physical stratigraphic tie points. Horizontal bands mark the glacial intervals according to the chronology of [3]. Panel B: same as the previous, but including the standard deviation for both analyses. Overlap between the two data series is well within the analytical uncertainties.

**References**

1. Herbert TD, Ng G, Cleaveland Peterson L. Evolution of Mediterranean sea surface temperatures 3.5–1.5 Ma: Regional and hemispheric influences. Earth and Planetary Science Letters. gennaio 2015;409:307–18.

2. Rossignol-Strick M, African monsoons, an immediate climate response to orbital insolation. Nature, 1983

3. Zanola E, Bonomo S, Incarbona A, Di Stefano A, Distefano S, Ferretti P, et al. High-resolution climate variability across the Piacenzian/Gelasian boundary in the Monte San Nicola section (Sicily, Italy). Quaternary Science Reviews. gennaio 2024;324:108469.

4. Robinson AR, Sellschopp J, Warn-Varnas A, Leslie WG, Lozano CJ, Haley PJ, et al. The Atlantic Ionian Stream. Journal of Marine Systems. 1 aprile 1999;20(1):129–56.

5. Incarbona A, Jonkers L, Ferraro S, Sprovieri R, Tranchida G. Sea Surface Temperatures and Paleoenvironmental Variability in the Central Mediterranean During Historical Times Reconstructed Using Planktonic Foraminifera. Paleoceanography and Paleoclimatology. 2019;34(3):394–408.
